# Label-Free Electrochemical Sensor Based on Manganese Doped Titanium Dioxide Nanoparticles for Myoglobin Detection: Biomarker for Acute Myocardial Infarction

**DOI:** 10.3390/molecules26144252

**Published:** 2021-07-13

**Authors:** Adel Al Fatease, Mazharul Haque, Ahmad Umar, Shafeeque G. Ansari, Yahya Alhamhoom, Abdullatif Bin Muhsinah, Mater H. Mahnashi, Wenjuan Guo, Zubaida A. Ansari

**Affiliations:** 1 Department of Pharmaceutics, College of Pharmacy, King Khalid University, Abha 62529, Saudi Arabia; ysalhamhoom@kku.edu.sa; 2 Centre for Interdisciplinary Research in Basic Sciences, Jamia Millia Islamia (Central University), New Delhi 110025, India; mazharulhaue23@gmail.com (M.H.); saansari@jmi.ac.in (S.G.A.); 3 Department of Chemistry, Faculty of Science and Arts, Najran University, Najran 11001, Saudi Arabia; 4 Promising Centre for Sensors and Electronic Devices (PCSED), Najran University, Najran 11001, Saudi Arabia; 5 Department of Pharmacognosy, College of Pharmacy, King Khalid University, Abha 61441, Saudi Arabia; ajmohsnah@kku.edu.sa; 6 Department of Pharmaceutical Chemistry, College of Pharmacy, Najran University, Najran 29613, Saudi Arabia; mhmahneshi@nu.edu.sa; 7 Institute of Surface Analysis and Chemical Biology, University of Jinan, Jinan 250022, China; chm_guowj@ujn.edu.cn

**Keywords:** acute myocardial infarction, myoglobin, metal oxide nanoparticles, electrochemical sensor, myoglobin sensor

## Abstract

A label free electrochemical sensor based on pure titanium oxide and manganese (Mn)-doped titanium oxide (TiO_2_) nanoparticles are fabricated and characterized for the sensitive detection of myoglobin (Mb) levels to analyze the cardiovascular infarction. Pristine and Mn-doped TiO_2_ nanoparticles were synthesized via the sol-gel method and characterized in order to understand their structure, morphologies, composition and optical properties. The structural properties revealed that the pure- and doped-TiO_2_ nanoparticles possess different TiO_2_ planes. FTIR studies confirm the formation of metal oxide nanoparticles by exhibiting a well-defined peak in the range of 600–650 cm^−1^. The values of the optical band gap, estimated from UV-Vis spectroscopy, are decreased for the Mn-doped TiO_2_ nanoparticles. UV-Vis spectra in the presence of myoglobin (Mb) indicated interaction between the TiO_2_ nanoparticles and myoglobin. The SPE electrodes were then fabricated by printing powder film over the working electrode and tested for label-free electrochemical detection of myoglobin (Mb) in the concentration range of 0–15 nM Mb. The fabricated electrochemical sensor exhibited a high sensitivity of 100.40 μA-cm^−2/^nM with a lowest detection limit of 0.013 nM (0.22 ng/mL) and a response time of ≤10 ms for sample S3. An interference study with cyt-c and Human Serum Albumin (HSA) of the sensors show the selective response towards Mb in 1:1 mixture.

## 1. Introduction

Acute Myocardial Infarction (AMI), a cardiovascular disease, is the first and most common cause for unexpected sudden death amid irreversible tissue damage or necrosis in the heart muscles [1]. It is known as one of the leading reasons of death in both low and middle income countries [2]. The cardiac biomarkers myoglobin (Mb) [3], cardiac troponin I (cTnI) [4] and cardiac troponin T (cTnT) [5] have been utilized to diagnose AMI.

Amongst all the biomarkers, Mb (a hemeprotein) has been widely recommended for early diagnosis of AMI owing to its small size (molecular weight of 16.8 kD) containing a single polypeptide chain (154 amino acids) and a porphyrin ring with central ferrous iron molecule [6]. Mb is released to the blood stream faster following the muscle damage, due to its presence in heart and skeletal muscle, and is also secreted from the kidney within 24 h of onset of symptoms [6,7,8]. After the onset of AMI, Mb releases into the blood stream within 30 min and its level rises up to 900 ng mL^−1^ within 6–12 h, returning to its normal physiological range of 30 to 90 ng mL^−1^ in the duration of 1.5 days [9,10,11]. Mb detection, therefore, became an important early diagnosis tool for AMI due to its high predictive value and good sensitivity [12], though Mb has no specificity.

Most frequent diagnostic methods are comprised of electrocardiographic (ECG) monitoring and testing of elevation of biomarkers. Recently, there is an enormous need to develop an alternative biochemical method for the diagnosis of AMI, as poor sensitivity and high associated costs create the demand for cheaper, faster and efficient methods of diagnosis to minimize damage due to AMI [13].

The reported methods for detecting Mb include optical fluorescence method [14], surface plasmon resonance method [15], mass spectroscopy [16], liquid chromatography [17], colorimetric method [18], molecular imprinting [19,20], immunoassay [21,22], chemiluminescence method [23] and electrochemical method [24,25]. However, the high cost of fabrication due to the use of expensive antibodies and noble metals such as Au, Ag or Pt limits its application. To overcome the problem, electrochemical based techniques can be the better alternative approach to estimate Mb content utilizing the presence of an electroactive heme center. We proposed a strategy for the sensing of Mb through electrochemical method using modified electrodes by Mn doped titanium oxide nanoparticles. Titanium nanoparticles were chosen owing to its biocompatible nature, as it is extensively used for biotechnological applications. Cyclic voltammetry and EIS was used to quantify the concentration of myoglobin in solution. The detection method was found to be faster (~10 ms) cheaper and easy to fabricate.

## 2. Experimental Details

### 2.1. Materials and Chemicals

Titanium tetra isopropxide (TTIP, 99%), isopropyl alcohol (99%), ethyl cellulose, butyl carbitol acetate, myoglobin (100684-32-0) and cytochrome c (9007-436) and Human Serum Albumin (HSA, 70024-90-7) were purchased from Sigma Aldrich, Delhi India. MnCl_2_·4H_2_O, were procured from Loba Chemicals, Delhi, India. NaH_2_PO_4_·2H_2_O (98%) and Na_2_HPO_4_ (99%), ammonium hydroxide (NH_4_OH, 28%), Nitric acid (HNO_3_) and sodium hydroxide (NaOH) were purchased from Fischer Scientific, Delhi, India. Highly resistive water (18 MΩ, Millipore) was used to prepare all the solutions.

### 2.2. Synthesis of TiO_2_ and Mn-Doped TiO_2_ Nanoparticles

Pristine and Mn doped TiO_2_ nanoparticles were synthesized using the sol-gel method following the method reported previously [26]. In separate reactions, 5 mL of TTIP (1.69 M) was dissolved in 10 mL isopropyl alcohol followed by dropwise addition of dopant precursor MnCl_2_·4H_2_O under constant stirring to obtain different doping concentration (0.26, 0.39 and 0.60 mM) which is equivalent to 13 × 10^17^, 20 × 10^17^ and 32 × 10^17^ atoms/cm^3^. The resulted gel was desiccated at 60 °C in an oven for 10 h followed by grinding to fine powder.

### 2.3. Characterizations of TiO_2_ and Mn-Doped TiO_2_ Nanoparticles

Crystallographic phase analysis of as-synthesized nanoparticles was carried out using powder X-ray diffraction (XRD) using Rigaku ultima IV diffractometer (CuK_α_ radiation, λ = 1.5418 Å) for Bragg angle (2θ) ranging between 20° to 80° at rate of 0.4 min^−1^ at room temperature. Particle size ‘*D*’ was estimated using the Deybe–Scherrer formula [27]:

(1) $$D=\frac{0.9\lambda }{\beta_{hkl}cos\theta }$$

where *D* is particle size; 0.9 is shape factor, *λ*= wavelength of CuK*_α_*, *β_hkl_* is full width at the half maximum of the maximum at Bragg angle *θ*.

The induced crystal strain during growth is estimated from W-H relation:

(2) $$\varepsilon =\frac{\beta_{hkl}}{4tan\theta }$$

and crystal dislocation density was estimated using the Smallman approach:

(3) $$\gamma \;=\;1/D^{2}$$

The surface morphology and topography were analyzed through field-emission electron microscopy (FESEM; Nova NanoSem, model 450) at an accelerating voltage of 10 keV. UV-Vis absorption measurement of as-synthesized nanoparticles and with different Mb concentrations was repeated by successive addition of Mb using U3900 spectrophotometer (Hitachi) for all the samples separately. The spectra were acquired in the wavelength range of 225 to 450 nm. For measurement, solution of as-synthesized nanomaterial was obtained by adding 50 µg of nanoparticle in 2 mL phosphate buffer (pH 7.4) which is treated as reference. This solution is then titrated by mixing required volume of Mb of solution in the existing solution. The analysis of surface functional properties was carried out for as-synthesized powders with and without 15 nM Mb from FTIR spectra in ATR mode acquired using Bruker’s Tensor 37 spectrophotometer.

### 2.4. Fabrication and Characterization of Mb Electrochemical Sensor

**Figure 1:**
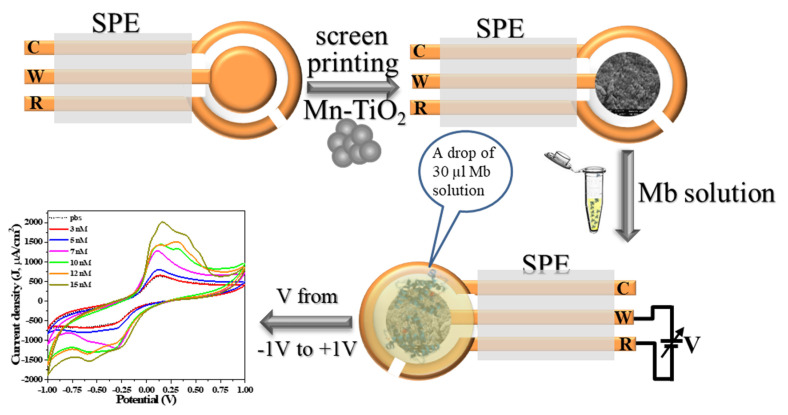
Process flow of electrochemical sensor fabrication and characterization.

The pre-fabricated three terminal Au-plated electrodes prepared on PCB were employed for screen printed electrodes (SPE). Figure 1 shows the fabrication process of SPE sensing electrode by printing the paste of as-synthesized nanoparticles over the working electrode (diameter of 4 mm) followed by electrochemical characterization. For printing, paste of as-synthesized nanoparticles was prepared using finely grinded powder in the agate-mortar and pestle by dropwise addition of organic binders that is mixture (70:30 ratios) of ethyl cellulose (EC) and butyl carbitol acetate (BCA). Thick film printed electrodes were allowed to settle for 10 min followed by drying at 60 °C for 4 h. Electrochemical activity of the electrodes was measured using cyclic voltammetry (CV) by sweeping the potential between −1.0 V to +1.0 V at a scan rate of 100 mV/S and performed using IVIUM’s potentiostat. CV curves were obtained in triplicate for each concentration (0–15 nM) and for all samples. Mb solutions of different concentrations from 0 nM to 15 nM were prepared in phosphate buffer solution (0.1 M, pH = 7.2) and characterized separately. For CV measurement, 30 µL of Mb solution was dropped over the SPE to cover all three electrodes (Figure 1). The graph of peak current against Mb concentration was utilized as calibration curve to calculate the sensitivity and estimate unknown concentration. Interference study was performed for sample S1, to understand the specificity and versatility of the sensor by mixing PBS solution of Mb and cyt-c (7 nM) and HSA (7 nM) separately and in 1:1 volumetric, keeping CV parameters as used for all samples. Further, to obtain the charge transfer kinetics, voltammograms were obtained for varied scan rate from 10 to 100 mV/S for 7 nM Mb concentration. All measurements were carried out at room temperature. An electrochemical impedance spectroscopy (EIS) was performed to analyze charge transfer characteristics for all fabricated sensor via potentiostat in the frequency range of 0.5 Hz–10^6^ Hz at amplitude of 50 mV.

## 3. Results and Discussion

### 3.1. Characterizations and Properties of Pristine and Doped tio_2_ Nanoparticles

**Figure 2:**
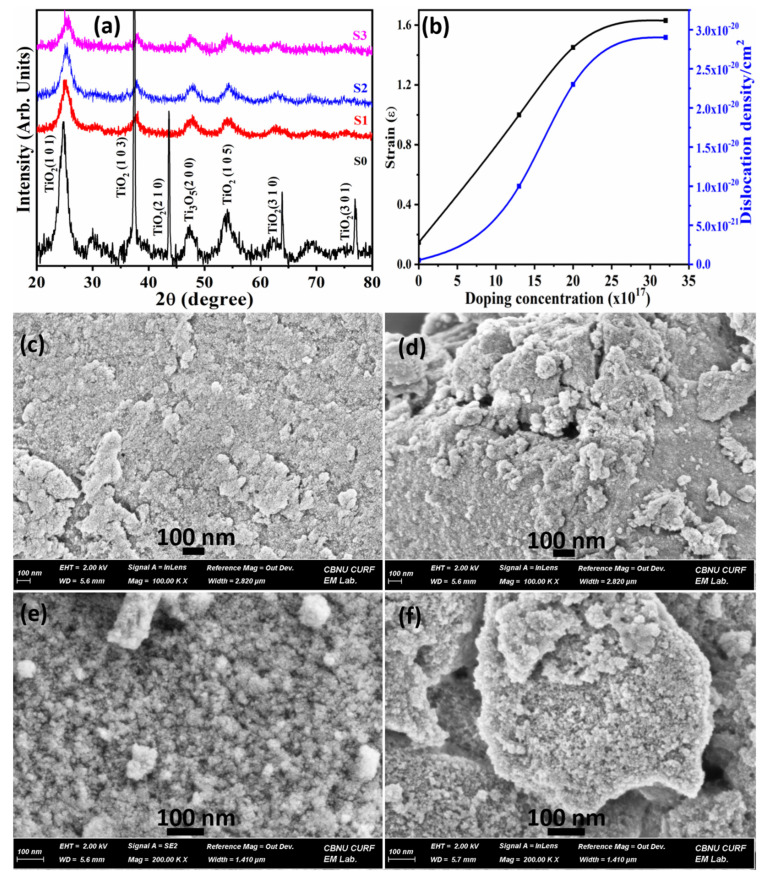
Typical (**a**) X-ray diffraction pattern (XRD), (**b**) Estimation of crystal strain and dislocation density and FESEM micrographs for (**c**) pristine TiO_2_ (**d**) TiO_2_–Mn (13 × 10^17^ atoms/cm^3^) (**e**) TiO_2_ –Mn (20 × 10^17^ atoms/cm^3^), and (**f**) TiO_2_–Mn (32 × 10^17^ atoms/cm^3^) samples.

Figure 2a depicts the XRD patterns of pure- and Mn doped-TiO_2_, which reveal peaks indicating growth of nanocrystals in different orientations. The black curve in Figure 2a presents pristine TiO_2_ that reveals peak related to TiO_2_ (101), (103), (210), (310) and (301) planes which are matched within Δd ≤ ±2% from the standard JCPDS files 89–4921 and 89-8304. There is no other peak than the two different phases of TiO_2_ (Figure 2a), which implies the purity of the grown nanocrystals. The Black curve in Figure 2a reveals a sharp peak along (103) suggesting preferential growth of nanocrystalline pristine TiO_2_. However, after doping (S1–S3) the slight shifting of XRD peaks is correlated to John-Teller distortion effect in the system and the reduction of peak intensity is due to reduced particle size. Substitution of Mn induced defects disturb the local ordering of the crystal structure, and distort crystallinity [28]. A peak of TiO_2_ (210) disappeared due to structural phase transition after doping. There is no peak related to Mn/Mn-TiO_2_ composite oxide indicates that Mn is replaced by Ti at the crystal sites and that free/unbound Mn is unavailable in the grown material. The average particle size as estimated from the Scherrer formula (Equation (1)) decreases in size after doping in comparison to pristine TiO_2_ (Table 1). The crystal strain and dislocation density were calculated using Equations (2) and (3), respectively. Both, the stress and dislocation density increase and then almost saturates with doping concentration as shown in Figure 2b due to the excess number of atoms, and defects on the amorphous grain boundary producing a stress field imposing a strain in the system [29].

Figure 2c–f depicts the FESEM images of as-synthesized pristine (S0) and Mn doped samples (S1-S3) that clearly suggest the change in morphology after doping. The images reveal fine spherical particles with uniform size are evenly distributed over the surface. However, doping results in the particle agglomeration that grow into large cluster-like structures, as observed for S3 samples (Figure 2f).

**Figure 3:**
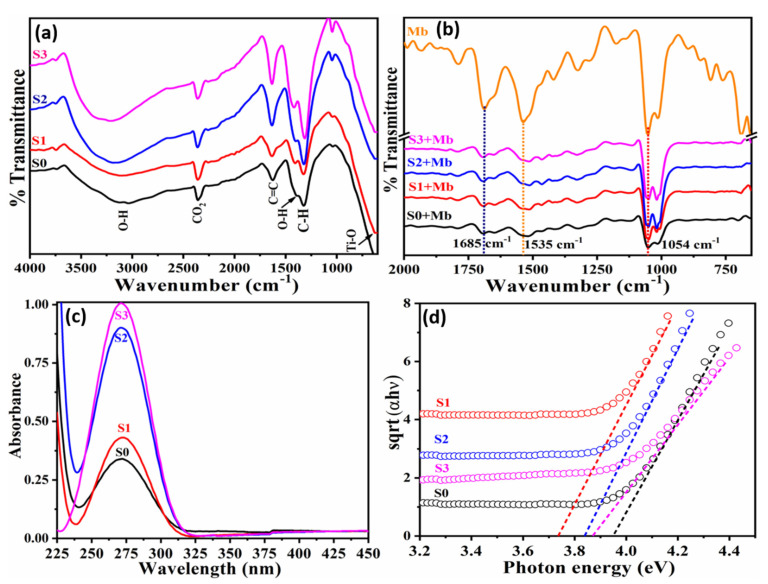
Typical FTIR spectra for the (**a**) as-synthesized materials and (**b**) materials interactions with Mb, (**c**) UV-Vis absorption spectra and (**d**) Tauc’s plot of undoped and Mn doped TiO_2_ nanoparticles [S0 = pristine Mn (13 × 10^17^ Mn atoms/cm^3^); S2 = TiO_2_ − Mn (20 × 10^17^ Mn atoms/cm^3^); S3 = TiO_2_ − Mn (32 × 10^17^ Mn atoms/cm^3^)].

FTIR spectra (Figure 3a) for pristine and Mn doped TiO_2_ as synthesized nanoparticles exhibit a broad band at 3220 cm^−1^ assigned to -OH stretching in the open atmosphere. Peak around 1630 cm^−1^ is that of C=C band. The band at 1410 cm^−1^ is due to O–H bending, and 1310 cm^−1^ is assigned to C–H. Band around 600–650 cm^−1^ is the finger print region of M-Ox that indicates the formation of TiO_2_. In general, the intensity of all the bands increased as a function of doping concentration. Figure 3b presents the FTIR spectra of the as-synthesized nanoparticles acquired in the presence of 15 nM Mb. FTIR spectra of Mb (orange curve) is obtained and treated as reference to estimate the changes of functionalized group over the nanoparticle surface in presence of 15 nM Mb. The Mb curve exhibits a band related to C–O stretching at 1054 cm^−1^ and that of N–H stretching at 1535 cm^−1^ in the amine II band. The peak at 1685 cm^−1^ appears due to C=O stretching whereas the peak around 800 cm^−1^ reflects Mb. The shifting of the peaks at 1685 cm^−1^ and 1535 cm^−1^ observed for pristine and doped TiO_2_ towards higher values suggest conjugation with Mb [30].

Figure 3c presents UV-Vis absorption spectra for Mn doped TiO_2_ nanoparticles. The peak absorption for pristine (S0) is at 272 nm, whereas for doped TiO_2_ (S1–S3) it is 271.5 nm, which indicates Mn replacing Ti in the crystal sites. The optical band gap estimated using Tauc’s plot (sqrt (αhν) vs. photon energy (eV)) shown in Figure 3d demonstrate low values of bandgap for doped TiO_2_ compared to that of pristine TiO_2_ and S1 revealing the minimum value, i.e., 3.2 eV of bandgap (Table 1). The reduction in the bandgap after doping is corelated to the increase of dislocation resulting into increased density of states. This result is in agreement with that of dislocation density estimated from XRD.

**Figure 4:**
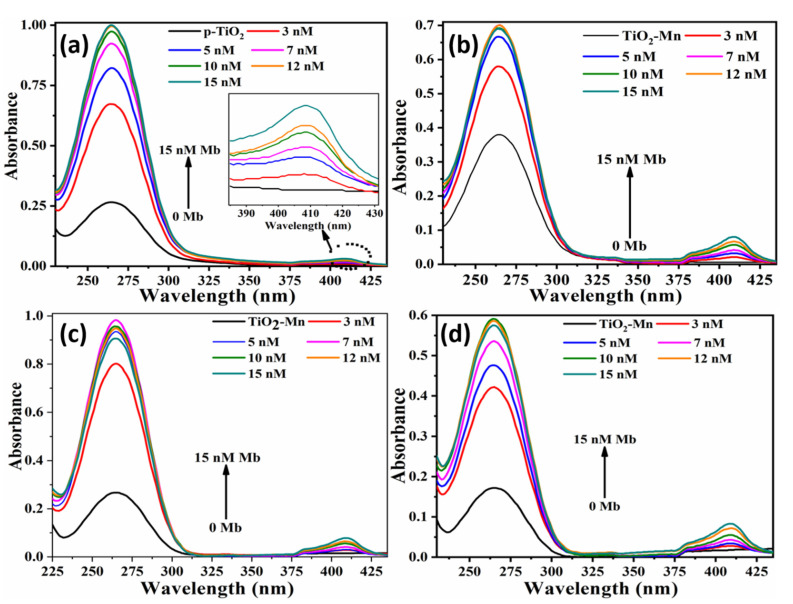
UV-Vis absorption spectra of pristine and Mn-doped TiO_2_ nanoparticles with various Mb concentrations (**a**) Pristine TiO_2_ (**b**) TiO_2_ − Mn (13 × 10^17^ atoms/cm^3^) (**c**) TiO_2_ − Mn (20 × 10^17^ atoms/cm^3^) (**d**) TiO_2_ − Mn (32 × 10^17^ atoms/cm^3^).

Figure 4a–d show the UV-absorption spectra for all, pristine (S0) and Mn doped TiO_2_ (S1–S3) obtained from the solution of as-synthesized nanoparticles with varying Mb concentrations from 3 nM–15 nM Mb. For measurements, 2 mL solution of nanoparticles (50 µg/2 mL) was prepared at pH 7.4 in which the proper volume of Mb solution was consequently added to attain the required Mb concentration. The absorption intensity and full width at half of maxima (FWHM) increases with increasing Mb concentrations, which indicates interaction between myoglobin molecules and TiO_2_ nanoparticles. The increase of intensity is due to the increased number of chromophores. Increase of FWHM versus Mb concentration implies the increase of oxidation attributed to the Fe oxidation with increasing Mb. Mb absorption showed a characteristic peak at 409 nm owing to heme which linearly increased with increasing concentration. UV-Vis results indicate the conjugation of Mb with nanoparticles [31].

### 3.2. Label-Free Mb Electrochemical Sensor Based on Pure- and Mn Doped-tio_2_ Nanoparticles

**Figure 5:**
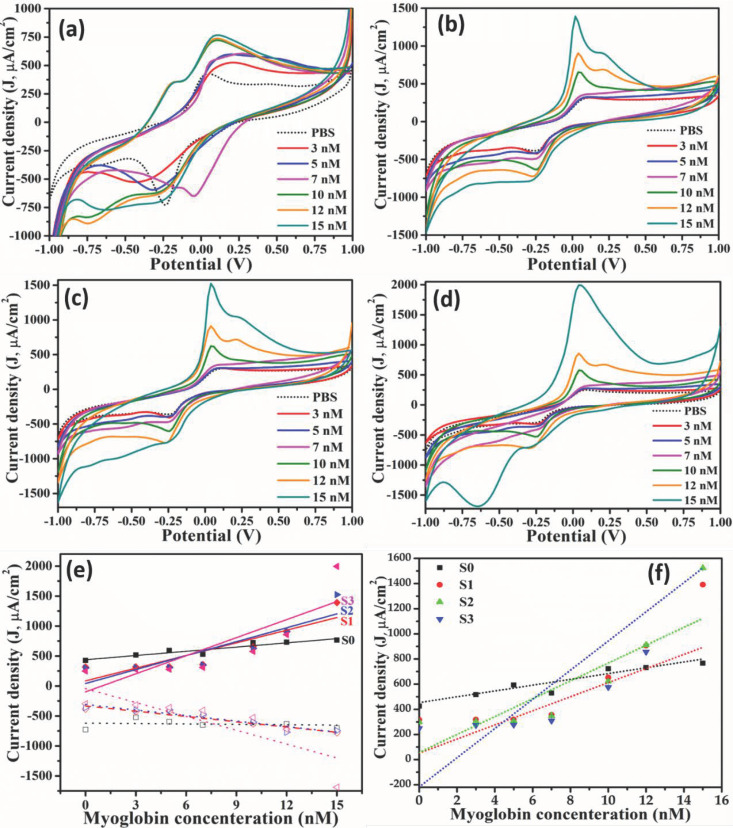
CV curves for different doping fractions: (**a**) S0: pristine TiO_2_; (**b**) TiO_2_–Mn (13 × 10^17^); (**c**) S1: TiO_2_–Mn (20 × 10^17^); (**d**) S3: TiO_2_–Mn (32 × 10^17^). (**e**) Oxidation peak current plotted as a function of Mb concentration used as calibration curve to estimate unknown Mb concentration; (**f**) calibration curve with error bar obtained from three different measurements.

The cyclic voltammogram for Mn doped TiO_2_ nanoparticles at different concentration of Mb is shown in Figure 5a–d, demonstrating the increase in oxidation peak current and decrease of reduction peak current for all fabricated sensors based on pristine and Mn doped TiO_2_ nanoparticles. The oxidation/reduction peak potential found at 0.18 V and -0.42 V, respectively, for pristine TiO_2_ implies the reversible reaction (Figure 5a). The oxidation and reduction peak potential were shifted towards the higher energy side with increasing Mb concentration, as the density of redox species increases at the electrode surface. For S1 samples (having 13 × 10^17^ atoms/cm^3^ of Mn), the oxidation and reduction peak potential was found at 0.1 V and −0.24 V, respectively, for 3 nM Mb (Figure 5b). Both the potentials are marginally shifted towards the low energy side (Figure 5b). However, for samples of TiO_2_ doped with 20 ×10^17^ atoms/cm^3^ of Mn (S2), they are found at 0.1 V and −0.24 V, respectively, with a slight shift in oxidation and reduction peak potential as seen in Figure 5c. For S3 samples, i.e., TiO_2_ doped with 32 × 10^17^ atoms/cm^3^, the oxidation and reduction peaks appeared at 0.08 V and −0.26 V, respectively, and are estimated from Figure 5d). The difference between oxidation and reduction in all cases are ≤0.60, suggesting the reversible nature of the reaction during sensing. However, the oxidation and reduction peak shifted towards low energy and the difference between redox peaks shrank for all samples at higher Mb concentrations. Additionally, the ratio of peak oxidation to reduction current is between 0.6 to 1.2 and is constant for repeated measurements, indicating the reaction reversibility. This behavior suggests that Mb conformation does not change and hence the sensor can be reused. The peak current density (J) was plotted against Mb concentration and treated as the calibration curve (Figure 5e) and the slope of the curve is estimated as the sensitivity of sensors, which is listed in Table 1. The increased sensitivity with doping concentration is correlated to the increased strain and density of states as reported earlier [32]. All the samples exhibited linear response in the characterized range, i.e., from 0–15 nM as evident from calibration curve (Figure 5e). In general, pristine (S0) and doped TiO_2_ (S1-S3), all show the low value of limit of detection (LOD) compared to that of reported values [33,34] with S3 exhibit the lowest LOD in comparison to all the studied samples (Table 2). The LOD for each sample was estimated from the ratio of standard deviation to the slope of calibration curve, i.e., (3.3 × standard deviation)/(slope of calibration curve). The obtained LOD is lowest possible than previous reported values (Bulko et.al., 2010; Tai et.al., 2002; Tripathi et.al., Wang et.al., 2014; Wang et.al., 2017; Yang et.al., 2017; Yue et.al., 2011). The error bar is shown in Figure 5f which shows the high degree of repeatability of the measurement indicating the repeatability of the device.

**Figure 6:**
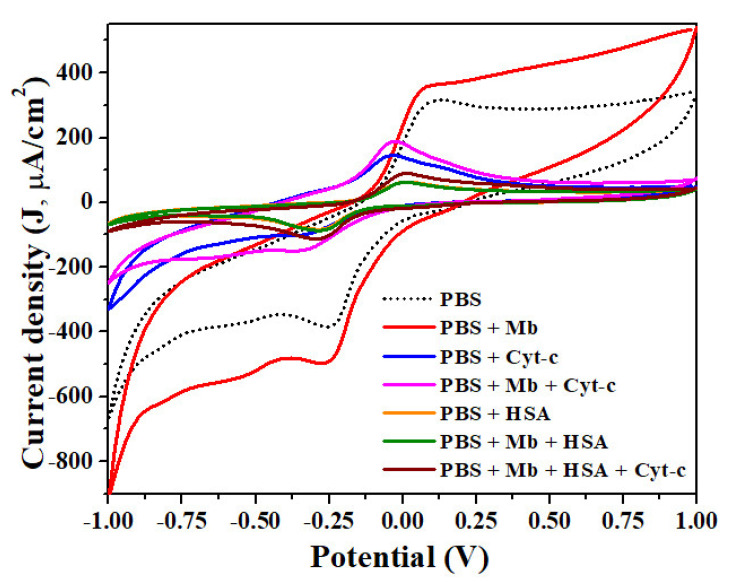
Interference study with Cyt-cand HSA for the fabricated sensor.

The interference study carried out with cyt-c and HSA, being a heme protein, is shown in Figure 6. It revealed different oxidation peak potentials for PBS, Mb, cyt-c and HSA as 0.1 V, 0.08 V, −0.04 V and 0.28 V, respectively. The peak oxidation and reduction potential for 1:1 mixture of cyt-c and Mb solution was −0.04 V and −0.36 V, respectively, and the redox peak potential for HAS and Mb in 1:1 ratio was +0.20 V and −0.28 V. The results imply that the developed sensors easily distinguish between two heme proteins as well as serum protein viz. cyto-c, HSA and Mb (Figure 6). The minimum peak current is observed for HAS even in the mixture of Mb + HSA (green curve) and Mb + cyt-c + HSA (wine curve). A slight increase in current density is observed when mixed with cyt-c and Mb in 1:1:1 ratio and is corelated to the presence of heme protein in the mixture. Additionally, Mb exhibited the maximum peak current density (red curve; Figure 6) reflecting the Mb detection selectivity of the developed sensors.

**Figure 7:**
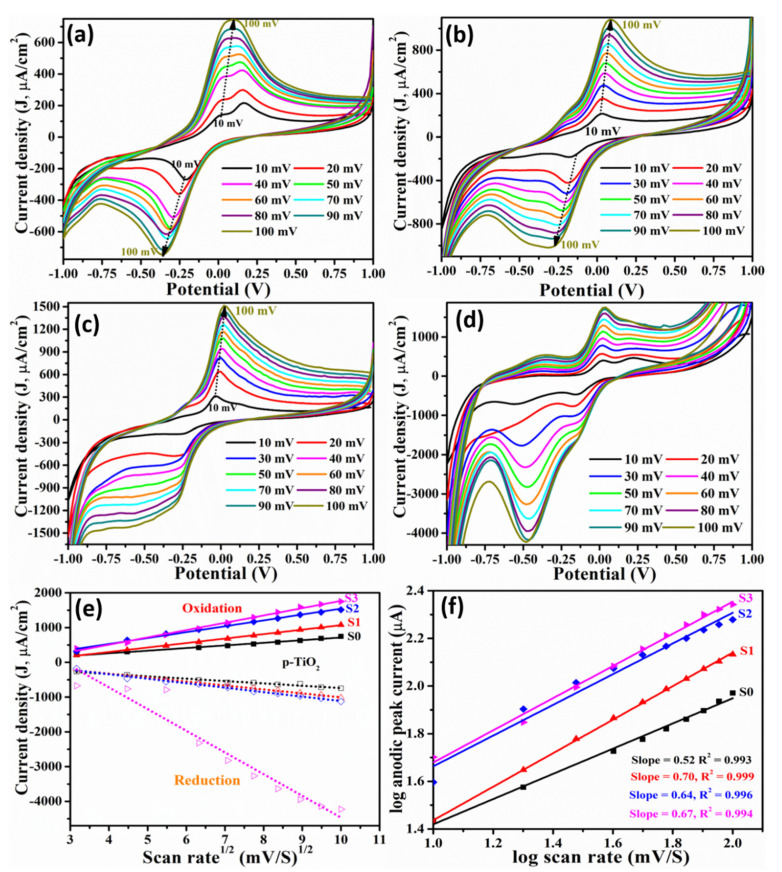
Effect of scan rate on the CV characteristics obtained at 7 nM of Mb for electrode made of (**a**) S0: pristine TiO_2_ (**b**) S1; TiO_2_ with 13 × 10^17^ Mn atoms/cm^3^ (**c**) S2: TiO_2_ with 20 × 10^17^ Mn atoms/cm^3^ (**d**) S3: TiO_2_ with 32 × 10^17^ Mn atoms/cm^3^ (**e**) peak current (both oxidation and reduction) versus square root of scan rate, and (**f**) log of peak current (oxidation) versus log of scan rate show linear response.

To analyze the reaction kinetics and process determination scan rate study was carried out at 7 nM Mb concentration (Figure 7a–d) and graphs of peak current (*v*^1/2^) versus scan rate (Figure 7e) and with natural log of scan rate (Figure 7f) exhibit a linear response for all samples suggesting a process to be diffusion-controlled. Figure 7a–d depicts scan rate dependent CV curves for different sensors of pristine and Mn doped TiO_2_ nanoparticles to evaluate their charge transfer characteristics. In general, a systemic and linear increase in reduction as well as oxidation peak current is observed with increasing scan rate and the redox potential shifted towards higher energy for all samples (Figure 7) with scan rate. The shifting of peak potential is correlated to the shift in the balance of the reaction. The increase in oxidation current with scan rate also indicates significant fast kinetics and ion transport for Faradic redox reactions [43]. The plot of peak current versus square root of the scan rate showed a linear relation in Figure 7e, and the characterized range indicates the fast ion transfer, reversible and diffusion-controlled reaction. The linearity implies sufficiently fast charge transfer to maintain the equilibrium. A plot of log of peak current versus log of scan rate depicted in Figure 7f, exhibit slope of 0.52 which is very close to the theoretical value of 0.5 for the diffusion-controlled process whereas a slope above 0.5 indicates that the process is driven by diffusion as well as adsorption.

**Figure 8:**
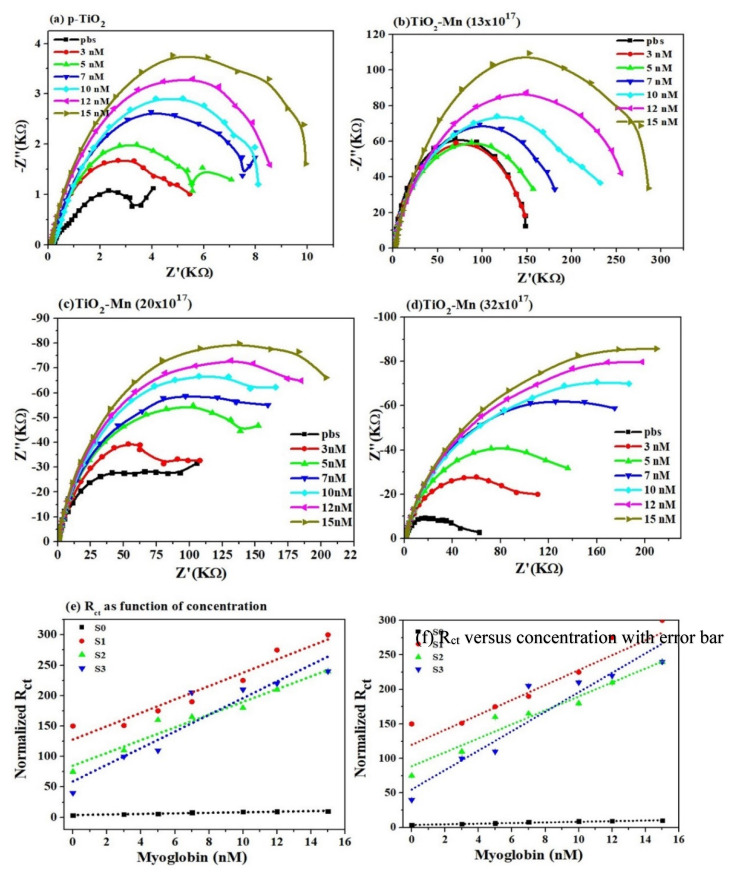
Series of Nyquist plots acquired for different Mb concentration for as synthesized TiO_2_ and Mn-doped TiO_2_ nanoparticles: (**a**) S0—pristine TiO_2_ (**b**) S1—TiO_2_-Mn (13 × 10^17^) (**c**) S2—TiO_2_-Mn (20 × 10^17^); (**d**) S3—TiO_2_-Mn (32 × 10^17^); (**e**) R*_ct_* values of Mb at 3–15 nM in PBS. (**f**) Error bar in R_ct_ values estimated from three measured cycles.

EIS curves obtained by plotting the imaginary part of impedance Z (Z”(KΩ)) versus the real part of Z ((Z’(KΩ)) for all the concentration in the frequency range 0.1 Hz to 1 × 10^6^ Hz reveal the increase in the value of charge transfer resistance R_ct_ at the surface interface as a function of Mb concentration for all the samples, as shown in Figure 8a–d. The semicircle at higher frequencies relates to the charge transfer resistance whereas the linear portion is like direct current behavior owing to the diffusion process. Values of charge transfer resistance (R_ct_) obtained from EIS curve plotted against Mb concentration show almost linear change in charge transfer (Figure 8e) and that is consistent for all the nanoparticles analyzed. The lowest R_ct_ value is observed for pristine TiO_2_ (S0), and that is increased with doping concentration; however, maximum linear variation is observed for 13 × 10^17^ atoms/cm^3^, resulting in enhanced sensitivity. The increase in the charge transfer resistance with doping is correlated to the presence of the high density of states as estimated from XRD results (Figure 2b). On the other hand, the increase of the charge transfer resistance with Mb concentration is associated to the dielectric and insulating features at the electrode/electrolyte at the electrode surface and electrolyte interface, as biomolecules are thin insulators. Therefore, high Mb concentration impedes the current flow due to effective increase of depletion region as evident from high R_ct_ values (Figure 8a–d) and higher energy required as evident from high value of redox potential at higher Mb concentration. Figure 8f reflects the error bar in the measured R_ct_ values obtained from three measurement cycles implies towards the reversibility of reaction and hence the repeatability.

Number of electrons involved in the reaction is estimated from the Randles–Sevcik equation:

(4) ip=0.4463.A.c.n32F32υDRT12

The results indicate that a single electron is involved for the redox reaction that takes place during the Mb sensing, which is expected as Fe^2+^ to Fe^3+^ or vice-versa conversion takes place in the process.

**Figure 9:**
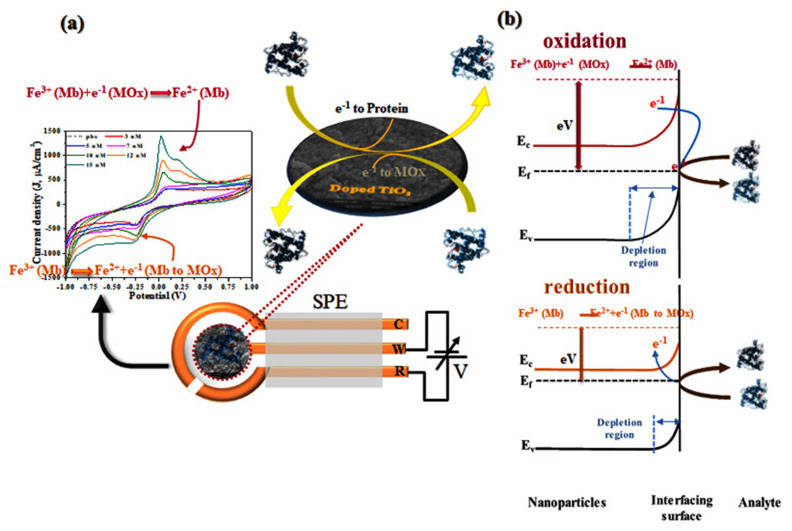
Proposed sensing mechanism (**a**) process of reaction mechanism (**b**) charge transfer characteristics.

Based on the experimental findings, a sensing mechanism is established in Figure 9, demonstrating the electron transfer mechanism during the redox reaction. Figure 9b demonstrates the change in the depletion region during the redox reaction wherein high energy requirement for oxidation and low energy for reduction process during sensing is required. Initially, Mb exists in an oxidized form due to presence of Fe ion (Fe^+2^ state); a known redox couple. While Mb sensing, as the applied potential at the electrode crosses above E^1/2^, the electron from the material is transferred to Mb converting from Fe^2+^ to Fe^3+^, reducing Mb and oxidizing TiO_2_. This process contributes to the increase of oxidative current and is shown in Figure 9a, where oxidized and reduced states of Mb are shown using the dark and light blue shades. The relative increase of the potential barrier at TiO_2_-Mb interface is shown in Figure 9b, wherein the electron is released from the material and adsorbed by the protein, inducing Mb reduction and increase of oxidation current. The resulting increase of electron depletion is shown in Figure 9b. While, during the negative sweep when the applied potential reaches to an equilibrium, an electron transfer takes place from Mb to TiO_2_ film wherein Fe^3+^ is converted to the Fe^2+^ state (Figure 9a); there is a light-blue to dark-blue transition, which reflects the releasing of an electron to the material and the reduction current is monitored [44]. The effective decrease in the electron depletion region as the result of injection of electrons to the conduction band of TiO_2_ is presented in Figure 9b. The increase in both the oxidation and reduction current as a function of Mb concentration is the result of the increase of Fe-ions on the sensor surface.

## 4. Conclusions

A nanostructure-based label free electrochemical sensor is fabricated using as-synthesized pristine and Mn doped TiO_2_ NPs. The structural and morphological characterization is confirmed through different techniques such as XRD, FTIR and UV-Vis spectroscopies, FESEM, etc. The as-synthesized nanopowders were applied for label free Mb sensing. XRD analysis confirms the growth of TiO_2_ with the crystallite size between 34–61 nm, whereas the presence of a peak around 600 cm^−1^ in the FTIR spectra confirms the synthesis of metal oxide (TiO_2_). The UV-Vis spectra in presence of various Mb concentrations revealed the interaction between nanoparticles and Mb. The electrochemical sensor characterized using CV suggested an increase in oxidation and a decrease in reduction peak is observed for all samples. The sensitivity was calculated using the slope of the linear region between the oxidation peak current and Mb concentration and it is observed increasing with increasing doping fraction. The S3 sample exhibited the highest sensitivity of 100.40 μA-cm^−2/^nM, with the lowest LOD of 0.013 nM (0.22 ng/mL). The scan rate dependent study shows the linear increase of current with Mb concentration as well as fast and reversible reaction and adsorption controlled at the interface of the electrode surface and electrolyte. The interference study demonstrates the distinct response for different analytes (Mb, cyt-c and HSA) and shows the versatility and specificity of the sensor. The EIS study revealed the consistent increase in R_ct_ value as a function of the Mb concentration. All the sensors exhibited linear response in the characterized range with less than 5% cycle to cycle variation, suggesting a high degree of repeatability and reusability of the sensors. The important outcome of the present study is the fabrication of a selective and reusable label free Mb sensor to detect cardiovascular infarction. This platform can be extended to other biomolecules/species for CVD and other diseases.

## Figures and Tables

**Table 1:** Materials and sensing properties of the fabricated sensors based on Mn-doped TiO_2_ nanoparticles.

TiO_2_ with Mn-Doping Concentration (Atoms/cm^3^)	Optical Band Gap (eV)	Grain Size (nm) *	Dislocation Density (×10^−18^ m^−2^)	Strain ε	Sensitivity μA-cm^−2/^nM	Diffusion Coefficient (D) (×10^−9^ cm^2^/s)
0 (S0)	3.95	39.38	0.005	0.14	23.43	1.62
13 × 10^17^ (SI)	3.73	61.30	0.010	1.00	70.44	3.17
20 × 10^17^(SII)	3.84	49.30	0.023	1.45	77.43	4.25
32 × 10^17^(SIII)	3.88	49.35	0.029	1.63	100.40	1.62

*: Estimated using Scherrer’s formula using XRD pattern.

**Table 2:** Comparison of the sensing properties of fabricated Mn-doped TiO2 nanoparticles biosensor with other reported biosensors.

Method	Matrix	Sample	Linear Range	LOD	Ref.
EIS	Ab-MYO/AuNps/APTES /ITO	Serum	10 ng mL^−1^–1 μg mL^−1^	2.7 ng mL^−1^	[35]
EIS	Anti-MYO/4-ATP SAM/Au	Saline,	350 ng mL^−1^–17.5 μg L^−1^	5.5 ng mL^−1^	[33]
DPV	MBA/AuNps/RGD/GRCOOH/GCE	Pork sample	0.0001–0.2 g L^−1^	26.3 ng mL^−1^	[34]
EIS	Anti-MYO/PtNP(PPy-PPa)-RGO/ITO	NA	10 ng mL^−1^–1 μg mL^−1^	4.0 ng mL^−1^	[36]
DPV	DSP/SAM/Au	Serum sample	17.8–1780 ng mL^–1^	9.8 ng mL^−1^	[37]
DPV	MIP/MWCNT/GCE	Serum	1 μg mL^−1^–0.1 mg mL^−1^	0.17 μg mL^−1^	[38]
SWV	MIP/PVC-COOH/Au-SPE	Serum	1.1–2.98 μg mL^−1^	2.25 μg mL^−1^	[39]
CV	Ti-NT/GCE	Serum	0.001–0.1 mg mL^−1^	1 μg mL^−1^	[13]
SWV	AuNp/DDAB/Anti- MYO/SPE	Serum sample	10–1780 ng mL^−1^	10 ng mL^−1^	[3]
CV	CS/MYOFe_3_O_4_@ SiO_2_/CILE	-	0.2–11.0 ng mL^−1^	0.18 ng mL^−1^	[40]
EIS	AuNp-PPy- PPa/RGO/APTES/ITO	Serum sample	10 ng mL^−1^–1 μg mL^−1^	1.49 ng mL^−1^	[12]
EIS	Ab-GO-MWCNT-Fe_3_O_4_	Serum urine	1–20000 ng mL^−1^	0.83 ng mL^−1^	[21]
Lateral flow Assay	NaYF_4_:Yb, Er@NaLuF_4_	Blood plasma	10–400 ng mL^−1^	0.21 ng/mL	[41]
Fluorescence	dabcyl [(E)-4-((4-(dimethylamino) phenyl) diazenyl)benzoic acid	PBS	0.1–5 ng/mL.	0.07 ng mL^−1^	[42]
CV/EIS	Mn doped TiO_2_ nanoparticles	PBS	3–15 nM	**S0**: 2.6 ng/mL (0.153 nM) **S1**:0.63 ng/mL (0.036 nM) **S2**:0.51 ng/mL (0.029 nM) **S3**:0.22 ng/mL (0.013 nM)	This work

## Data Availability

Not applicable.

## Sample Availability

Samples of the compounds are available from the authors.
